# Morphometric Changes to Corneal Dendritic Cells in Individuals With Mild Cognitive Impairment

**DOI:** 10.3389/fnins.2020.556137

**Published:** 2020-12-09

**Authors:** Cirous Dehghani, Shaun Frost, Rajiv Jayasena, Christopher Fowler, Colin L. Masters, Yogesan Kanagasingam, Haihan Jiao, Jeremiah K. H. Lim, Holly R. Chinnery, Laura E. Downie

**Affiliations:** ^1^Department of Optometry and Vision Sciences, University of Melbourne, Parkville, VIC, Australia; ^2^CSIRO, Australian e-Health Research Centre (AEHRC), Parkville, VIC, Australia; ^3^Discipline of Optometry, University of Canberra, Canberra, ACT, Australia; ^4^CSIRO, Australian e-Health Research Centre (AEHRC), Floreat, WA, Australia; ^5^The Florey Institute of Neuroscience and Mental Health, University of Melbourne, Parkville, VIC, Australia; ^6^Optometry and Vision Science, College of Nursing and Health Sciences, Flinders University, Adelaide, SA, Australia

**Keywords:** dendritic cell, peripheral nervous system, confocal microscopy, sensory nerves, central nervous system, IVCM, peripheral nerves, immune cell

## Abstract

**Purpose:**

There has been increasing interest in identifying non-invasive, imaging biomarkers for neurodegenerative disorders of the central nervous system (CNS). The aim of this proof-of-concept study was to investigate whether corneal sensory nerve and dendritic cell (DC) parameters, captured using *in vivo* confocal microscopy (IVCM), are altered in individuals with mild cognitive impairment (MCI) and Alzheimer’s disease (AD).

**Methods:**

Fifteen participants were recruited from the Australian Imaging Biomarkers and Lifestyle (AIBL) study in Melbourne, VIC, Australia. The cohort consisted of cognitively normal (CN) individuals (*n* = 5), and those with MCI (*n* = 5) and AD (*n* = 5). Participants underwent a slit lamp examination of the anterior segment, followed by corneal imaging using laser-scanning *in vivo* confocal microscopy (IVCM) of the central and inferior whorl regions. Corneal DC density, field area, perimeter, circularity index, aspect ratio, and roundness were quantified using Image J. Quantitative data were derived for corneal nerve parameters, including nerve fiber length (CNFL), fiber density (CNFD), branch density (CNBD), and diameter.

**Results:**

Corneal DC field area and perimeter were greater in individuals with MCI, relative to CN controls, in both the central and inferior whorl regions (*p* < 0.05 for all comparisons). In addition, corneal DCs in the whorl region of MCI eyes had lower circularity and roundness indices and a higher aspect ratio relative to CNs (*p* < 0.05 for all comparisons). DC density was similar across participant groups in both corneal regions. There was a trend toward lower quantitative parameters for corneal nerve architecture in the AD and MCI groups compared with CN participants, however, the inter-group differences did not reach statistical significance. Central corneal nerve diameters were similar between groups.

**Conclusion:**

This study is the first to report morphological differences in corneal DCs in humans with MCI. These differences were evident in both the central and mid-peripheral cornea, and in the absence of significant nerve abnormalities or a difference in DC density. These findings justify future large-scale studies to assess the utility of corneal IVCM and DC analysis for identifying early stage pathology in neurodegenerative disorders of the CNS.

## Introduction

Alzheimer’s disease (AD) is the most common form of irreversible dementia; it is estimated to affect about 40 million people worldwide (Wu et al., 2017). AD is a progressive neurodegenerative disorder characterized by brain atrophy associated with the presence of Beta-amyloid (Aβ) plaques and tau tangles (Ballard et al., 2011). The condition poses a major global public health and economic challenge and is one of the major unmet medical needs in neurology (World Alzheimer Report, 2015; Scheltens et al., 2016).

Alzheimer’s disease is diagnosed clinically by episodic memory problems that progress to a gradual global decline of higher cognitive function. AD is preceded by mild cognitive impairment (MCI), which represents an intermediate clinical state between the cognitive changes of aging and the earliest features of AD (Petersen et al., 1999). While a definitive diagnosis of AD is made on the basis of autopsy brain findings (Ballard et al., 2011), neuroimaging studies, including magnetic resonance imaging (MRI), and positron emission tomography (PET), are common clinical diagnostic methods. Cerebrospinal fluid biomarkers and amyloid PET, which can detect earlier AD pathology, have revealed subtle subclinical pathological changes that occur before the clinical decline (Villemagne et al., 2011, 2013; Vos et al., 2013). However, these advanced procedures are impractical for routine use due to their cost and invasiveness. The United States National Institute of Aging and Alzheimer’s Association have emphasized a need to identify reliable biomarkers that can identify individuals at highest risk for cognitive decline or progression from MCI to AD (Albert et al., 2011). Developing accessible, non-invasive and sensitive biomarkers of early stage disease will provide an essential foundation for both investigating new treatments and enabling earlier disease intervention (Vos et al., 2013; Scheltens et al., 2016).

In the quest for a practical, early stage biomarker of AD, the eye and its neural network have been studied, with a particular focus on the retina – as a developmental outgrowth of the brain (Lim et al., 2016; Hadoux et al., 2019; Szegedi et al., 2020). Retinal axonal and neural degeneration (Lim et al., 2016; Cheung et al., 2017), and the presence of Aβ in retinal tissues (Lim et al., 2016; van Wijngaarden et al., 2016), have been reported in individuals with AD. Researchers have also considered the potential utility of markers related to assessing retinal blood flow and vascular integrity (Frost et al., 2013a; Feke et al., 2015), pupil responses (Prettyman et al., 1997; Fotiou et al., 2000; Frost et al., 2013b) and Aβ signatures in the crystalline lens (Goldstein et al., 2003; Kerbage et al., 2015). However, relative to the posterior ocular segment there has been less attention on potential anterior eye biomarkers of AD (Dehghani et al., 2018). The anterior eye, and in particular the cornea, has the advantage of being highly accessible for non-invasive imaging and evaluation.

The cornea is one of most highly innervated tissues in the body. It is supplied by sensory nerve axons originating from the trigeminal ganglion that comprise part of the peripheral nervous system (PNS) (Marfurt et al., 2010; He and Bazan, 2016). The corneal nerves enter the tissue radially, branching progressively as they course toward the apical layers of the corneal epithelium. Toward the center of the cornea, the axons form a unique whorl-like pattern, which in humans is typically located slightly inferior to the corneal apex. This whorl-like pattern serves as a useful landmark for identifying consistent anatomical locations between individuals. Recently, there has been interest in using non-invasive corneal nerve imaging, using *in vivo* confocal microscopy (IVCM) to examine the integrity of the PNS in central nervous system (CNS) neurodegenerative diseases (Vieira et al., 2015; Podgorny et al., 2016; Bitirgen et al., 2017; Mikolajczak et al., 2017; Petropoulos et al., 2017; Ponirakis et al., 2019). To date, there has only been one study evaluating central corneal nerves in individuals with dementia (Ponirakis et al., 2019). The cornea, in particular its basal epithelium, is also populated by resident immune cells, known as dendritic cells (DCs) (Yamagami et al., 2005; Mastropasqua et al., 2006). In addition to their role as immune sentinels, which bridge innate and adaptive immune responses, (Hamrah et al., 2003; Hattori et al., 2016), corneal DCs also contribute to maintaining corneal nerve homeostasis (Gao et al., 2016). Studies in mice have demonstrated that corneal DC populations, analogous to the immune cells visible using IVCM in humans, are morphologically altered at an early stage of frontotemporal dementia, prior to the onset of corneal nerve degeneration (Jiao et al., 2019). Despite this, there have not yet been any clinical studies investigating whether corneal DCs are altered in people with dementia. The aim of this study was to investigate whether sensory nerve and DC parameters in both the central and mid-peripheral cornea, captured non-invasively using IVCM, are altered in individuals with AD and MCI.

## Materials and Methods

This cross-sectional study received ethics approval from St Vincent’s Hospital (Melbourne) Human Research Ethics Committee (HREC) (Ref: HREC-A 028/06), was endorsed by the CSIRO Health and Medical Research Ethics Committee (RR 5/2018) and was prospectively registered with the University of Melbourne HREC (ID: 1852006). All participants provided written informed consent to participate, in accordance with the Declaration of Helsinki.

### Study Participants

As a proof-of-concept study, no formal *a priori* sample size calculation was performed. A convenience sample of 15 participants was recruited from the Australian Imaging Biomarkers and Lifestyle (AIBL) study, Melbourne, VIC, Australia (Ellis et al., 2009), between June 2018 and August 2018.

To be eligible to participate, potential participants were required to: be aged ≥60 years, have provided written informed consent to participate, and be enrolled in the AIBL study with a current diagnosis of one of: Alzheimer’s disease (AD, *n* = 5), mild cognitive impairment (MCI, *n* = 5) or cognitive normal (CN, *n* = 5). As adopted in the AIBL study (Ellis et al., 2009), participant assignment to one of these three health subcategories was made on the following grounds: (i) individuals meeting the criteria for based on the NINCDS-ADRDA (National Institute of Neurological and Communicative Disorders and Stroke–Alzheimer’s Disease and Related Disorders Association) (McKhann et al., 1984), (ii) individuals meeting the criteria for MCI (Petersen et al., 1999; Winblad et al., 2004), and (iii) CN control individuals.

Potential participants meeting any of the following criteria were ineligible to participate: history of corneal trauma or surgery (with the exception that cataract surgery did not preclude enrolment, unless the surgery occurred in the 12 months prior to enrolment), history of any ocular or systemic condition known to affect corneal health (e.g., contact lens wear, diabetes), any concurrent ocular disease, infection or inflammation, history of neuropathy due to any other causes (e.g., alcoholism, autoimmune disorders) and a known allergy to any eye drop formulations.

### Study Procedures

Study procedures were performed at The Florey Institute of Neuroscience and Mental Health (Oak Street site), Parkville, VIC, Australia. Baseline demographic and clinical information (e.g., age, gender, medical, and ophthalmic histories) were captured from each participant.

#### Neuropsychological Evaluation and Neuroimaging

Comprehensive neuropsychological tests were performed for all participants as part of the AIBL study, including the Mini-Mental State Exam (MMSE) to assess cognitive function (Folstein et al., 1975) and Clinical Dementia Rating Scale (CDR). Details of the full testing protocol has been comprehensively described elsewhere (Ellis et al., 2009).

Amyloid beta positron emission tomography (Aβ-PET) neuroimaging was also performed as part of the AIBL study, as previously described (Rowe et al., 2010). The Centiloid values were derived for all participants and the Centiloid scale for the global measure of neocortical amyloid burden (NAB) was utilized (Klunk et al., 2015).

#### Ocular Health Screening

As part of the eligibility assessment, participants had a bilateral ocular health screening assessment, involving a slit lamp examination using white light and sodium fluorescein (to assess for corneal staining) to confirm the absence of clinically significant ocular surface pathology (e.g., dry eye disease), as determined by clinical judgment (LED and CD).

#### Corneal *in vivo* Confocal Microscopy (IVCM)

Participants underwent corneal laser-scanning IVCM (Heidelberg Retina Tomograph-3 with the Rostock Corneal Module, Heidelberg Engineering, Germany). This device generates two-dimensional images, 384 × 384 pixels in size and covering a corneal area of 400 × 400 μm when used with a 63× objective lens.

The cornea of the dominant hand side of the participant was anesthetized with one drop of non-preserved topical 0.4% benoxinate hydrochloride (Novartis, United States). Participants were instructed to fixate on a near target with the contralateral eye. The same targets were used to guide fixation for all participants. Images (400 × 400 μm) of the anaesthetized cornea were captured at the level of the sub-basal nerve plexus (SBNP) in two regions: the apex (central cornea) and inferior whorl (mid-peripheral cornea). Manual focussing and the section-scan mode were used to capture >50 unique images in each region of interest. Illumination was set at automatic brightness for all image captures. No post-capture image enhancements were performed. As noted by Patel and McGhee (2009), this image capture method is essential to the validity of the corneal nerve thickness parameter quantification (Patel and McGhee, 2009).

Images that were out of focus, not in a consistent plane or showed artifactual compression lines were excluded from the analysis set. To ensure an unbiased selection of analyzed the SBNP images, eight non-overlapping images and three non-overlapping images were randomly selected and analyzed, by a masked operator, from the central cornea and inferior whorl regions, respectively, for each participant. In total, 120 images of the central cornea and 39 images of the inferior whorl were analyzed in this study. For the central cornea, this level of sampling has been shown to provide average values within 30% of the true mean value, 95% of the time (Vagenas et al., 2012; De Silva et al., 2017). No post-capture image enhancements were made to any of the images.

A repeat slit lamp examination was performed after the IVCM procedure to ensure an absence of any ocular surface trauma from the procedure.

#### IVCM Image Analysis

The IVCM image analyses were performed by a masked researcher, naïve to the participant group.

##### Corneal nerves

Nerve parameters were quantified using the automated ACCMetrics software (Dabbah et al., 2010; Dehghani et al., 2014) for central: corneal nerve fiber length (CNFL), corneal nerve fiber density (CNFD), corneal nerve branch density (CNBD), and corneal nerve total branch density (CTBD). CNFL is defined as the total length of all nerve fibers in the image capture frame per unit area (mm/mm^2^), CNFD is defined as the total number of main fibers divided by the area of the image frame (fibers/mm^2^), CNBD is defined as the total number of main nerve branches, being branches that stem from a nerve fiber divided by the area of the image frame (branches on main fiber/mm^2^) and CTBD is defined as the total number of branches within the area of the image frame (total branches/mm^2^). Only CNFL was quantified in the inferior whorl region. For each parameter, the average value from the eight (central) and three (inferior whorl) non-overlapping images was taken as the representative value for each participant.

To quantify central corneal nerve diameter, the two thickest primary nerves with no branches were selected in each image. Images were processed using ImageJ (v1.47, NIH, United States). First, uneven background illumination was corrected using “Subtract Background,” followed by “Median Filter” to smooth nerve edges. Eight-bit images were inverted and the corneal nerve diameter was determined using the “Diameter” plugin (Fischer et al., 2010). Measurements were performed by drawing a series of three perpendicular line segments traversing each nerve, in order to determine a greyscale intensity profile. Each region was sampled five times, to give a total of thirty nerve diameter readings per image. The individual diameter values were averaged to yield a single representative nerve diameter measurement for each image. With a total of eight IVCM images collected per participant, values were further averaged to produce a single representative diameter value for each participant.

##### Corneal dendritic cells (DCs)

The density and shape characteristics of presumed central corneal DCs, residing in the epithelium, were quantified using the same images as for the SBNP analysis. DCs were identified as bright, dendriform cells at the level of the SBNP. DC density (cells/mm^2^) was quantified using the cell counter plugin of ImageJ, with the overall density derived from averaging the density from the eight non-overlapping images in the central cornea and three non-overlapping images in the whorl region. To exclude the potential influence of inter-observer variability, the same masked observer (with expertise in IVCM image acquisition, interpretation, and analysis) performed all DC analyses.

Dendritic cell morphology was quantified in ImageJ, using the “Polygon Selections” feature tool [to manually connect points comprising the outermost aspects of the dendrites of individual DCs (Kheirkhah et al., 2015)] and the Analyze – Measure function, to quantify the following shape descriptors: field area (μm^2^), field perimeter (μm), circularity, aspect ratio (AR), and roundness. The circularity function calculates object circularity using the formula: *circularity = 4π (area/perimeter^∧^2)*; a circularity value of 1.0 indicates a perfect circle, and as the value approaches 0.0, it indicates an increasingly elongated polygon. The AR is derived as the ratio of the distance of the major axis divided by the minor axis. Roundness is calculated as *4 × area/(π× major_axis^∧^2*). These shape descriptors were quantified for 20 unique corneal DCs per participant. To minimize any potential bias in DC selection, cells were consecutively quantified from the top left hand corner of randomly selected images, until the required number of samples was achieved. Between four and eight separate non-overlapping central corneal image regions were required to achieve the nominated number of DCs across the study population. For each DC shape descriptor, an average value was derived per participant. For DC field area, a “per cell” analysis, involving the analysis of 100 individual corneal DCs per group, is also presented to show the cell distribution characteristics.

### Statistical Analyses

Statistical analyses were performed in GraphPad Prism (version 7.0; GraphPad Software, Inc., La Jolla, CA, United States). Descriptive statistics are used to summarize participant demographic and clinical characteristics. For quantitative continuous data, normality was tested using the D’Agostino and Pearson omnibus test. For continuous parametric data, one-way analysis of variance (ANOVA) tests were performed to assess for inter-group differences; in cases of statistically significant outcome on the ANOVA, a Tukey’s *post-hoc* test was performed to compare groups. For continuous non-parametric data, a Kruskal–Wallis test was performed to assess for inter-group differences, with *post-hoc* testing using Dunn’s Multiple Comparisons Test when appropriate. For statistical significance, the alpha was set at 0.05.

## Results

### Participant Characteristics

All enrolled participants (*n* = 15) completed the study. The corneal IVCM procedure was well tolerated in all participants, with no adverse events. Central corneal IVCM images were acquired from all participants. Images of the inferior whorl were successfully captured for 13 of 15 participants (*n* = 5 CN, *n* = 4 MCI, *n* = 4 AD).

The demographic and clinical characteristics of the study participants are summarized in Table 1. Participants in each group were of similar age. None of the study participants were taking medications known to affect corneal integrity. Furthermore, none of the participants had known allergic eye disease or atopy, dry eye disease or systemic diseases known to affect corneal dendritic cell status, including diabetes or rheumatoid arthritis. A summary of the categories of concomitant medications taken by participants is provided in Supplementary Table 1.

**TABLE 1:** Demographic characteristics of the study participants.

	**CN (*n* = 5)**	**MCI (*n* = 5)**	**AD (*n* = 5)**
Gender (male/female)	2/3	4/1	4/1
Age (years)	74.0 ± 3.5	74.6 ± 7.5	80.4 ± 4.3
MMSE score (/30)	28.6 ± 0.5	25.4 ± 3.6	25.6 ± 1.1
PET positive for beta-amyloid (Aβ) (yes/no)	3/2	4/1	5/0
Centiloid	66.7 ± 53.5	89.2 ± 48.6	91.1 ± 27.7

*Data are presented as mean ± SD, or as counts for categorical variables. MMSE, Mini Mental State Examination; PET, positron emission tomography.*

There was no significant inter-group difference in the MMSE score, although cognitive normal (CN) participants trended toward a higher level of performance on this cognitive test. At the most recent assessment of cognitive function prior to eye testing, all CN participants had a CDR of 0, all MCI participants had a CDR of 0.5 and of the five AD participants, four had a CDR of 0.5 and one had a CDR of 1.0. All AD participants, four MCI and three CN participants were positive for Aβ on positron emission tomography (PET). Centiloid values were found to be high in 5/5, 4/5, and 3/5 AD, MCI, and CN participants, respectively. The mean Centiloid values did not differ significantly among the three groups (*p* = 0.64).

### Corneal Characteristics

For all study participants, no clinically significant corneal pathology was noted on the baseline slit lamp examination.

#### Nerve Architecture

As summarized in Table 2, there was a trend toward lower quantitative corneal SBNP parameters in the MCI and AD groups, compared with CN participants in both the central and inferior whorl regions, however, none of the comparisons reached statistical significance (*p* > 0.05 for all comparisons). The average central corneal nerve diameter of the thickest primary nerves was also similar amongst the study groups (*p* > 0.05).

**TABLE 2:** Corneal sub-basal nerve plexus parameters.

	**CN (*n* = 5)**	**MCI (*n* = 5)**	**AD (*n* = 5)**	***p*-value**
**Central area**				
CNFL (mm/mm^2^)	17.3 ± 4.6	16.2 ± 2.6	13.7 ± 6.0	0.48
CNFD (fibers/mm^2^)	27.8 ± 8.5	24.4 ± 3.9	22.5 ± 11.0	0.60
CNBD (number/mm^2^)	53.3 ± 20.5	43.9 ± 26.0	35.2 ± 18.9	0.45
CTBD (total branches/mm^2^)	75.9 ± 27.9	67.5 ± 40.4	47.3 ± 21.9	0.36
Thickest nerve diameter (μm)	5.28 ± 0.17	5.08 ± 0.11	5.25 ± 0.20	0.66
**Inferior whorl^∧^**				
CNFL (mm/mm^2^)	15.9 ± 4.5	10.4 ± 4.3	13.8 ± 3.3	0.19

*CNFL, total length of all nerve fibers in the image capture frame per unit area; CNFD, total number of main fibers divided by the area of the image frame (fibers/mm^2^); CNBD, total number of main nerve branches, being branches that stem from a nerve fiber, divided by the area of the image frame (branches on main fiber/mm^2^); CTBD, total number of branches within the area of the image frame (total branches/mm^2^). ^∧^Inferior whorl data were available for *n* = 5 cognitive normal (CN), *n* = 4 mild cognitive impairment (MCI), and *n* = 4 Alzheimer’s disease (AD) participants.*

#### Dendritic Cell Density and Morphology

##### Central cornea

**FIGURE 1:**
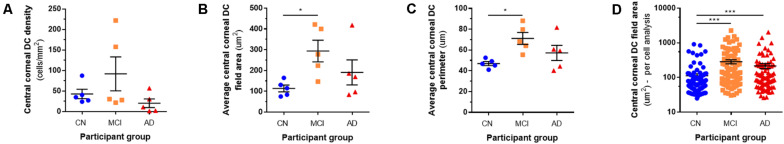
Central corneal dendritic cell parameters. Central corneal DC density **(A)** was similar amongst the cognitive normal (CN), mild cognitive impairment (MCI), and Alzheimer’s disease (AD) participants groups. On average, central corneal DCs had a larger field area **(B)** and perimeter **(C)** in MCI eyes, relative to CNs. In the “per cell” analysis, central corneal DCs from CNs had significantly smaller field areas than cells from individuals with MCI and AD **(D)**. *p < 0.05, ***p < 0.001.

Figure 1 summarizes data relating to central corneal DC density and morphology. Although there were no significant inter-group differences for central DC density (Figure 1A), average DC field area (Figure 1B: mean ± SD, MCI: 294.0 ± 116.6 versus CN: 114.0 ± 36.3 μm^2^, *p* = 0.04) and perimeter (Figure 1C: MCI: 71.1 ± 12.9 versus CN: 46.9 ± 4.3 μm, *p* = 0.04) were significantly greater in MCI participants relative to CN controls. The “per cell” analysis of DC field area (Figure 1D) also showed that, as a population, central corneal DCs from CNs had significantly smaller field areas than cells from individuals with MCI and AD (*p* < 0.0001).

**FIGURE 2:**
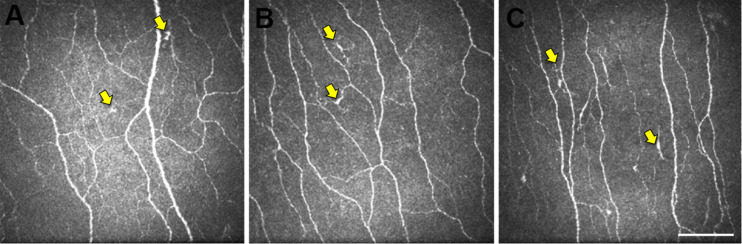
Representative central corneal IVCM images. Images of the central corneal sub-basal nerve plexus from participants in the CN **(A)**, MCI **(B)**, and AD **(C)** groups. The epithelial DCs (yellow arrows) are smaller and less stratified in CN eyes **(A)** relative to the larger more stratified morphology of DCs in MCI and AD eyes. Scale bar: 100 μm, applies to all images.

Representative central corneal IVCM images from a participant in each study group, taken at the level of the corneal SBNP, are provided in Figure 2. These images provide visualization of the quantified phenotypic difference in central corneal DCs, which were larger and more stratified in the MCI group, compared with DCs from CNs. Furthermore, representative IVCM images of the central corneal region, for each study participant, are provided in Supplementary Figure 1. DC shape parameters relating to circularity, AR and roundness did not show any significant inter-group differences (Supplementary Figure 2).

##### Inferior whorl

**FIGURE 3:**
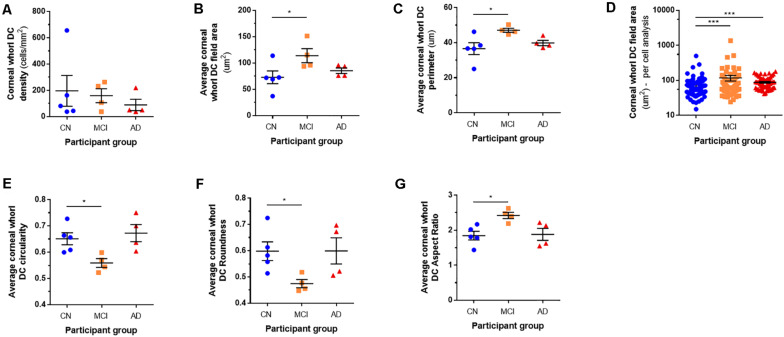
Corneal whorl dendritic cell parameters. Corneal DC density in the mid-peripheral inferior whorl region **(A)** was similar amongst the cognitive normal (CN), mild cognitive impairment (MCI), and Alzheimer’s disease (AD) participant groups. On average, the corneal DCs had a larger field area **(B)** and perimeter **(C)** in MCI eyes, relative to CNs. In the “per cell” analysis, DCs from CNs had smaller field areas than cells from individuals with MCI and AD **(D)**. In addition, DCs in the inferior whorl region of MCI eyes were less circular **(E)**, less round **(F)**, and had a higher aspect ratio **(G)** than DCs in CNs. *p < 0.05, ***p < 0.001.

Figure 3 summarizes data relating to corneal DC density and morphology in the inferior whorl region. Representative IVCM images of the corneal inferior whorl region, for each study participant, are provided in Supplementary Figure 3.

Although there were no significant inter-group differences for DC density (Figure 3A), all other morphometric parameters were significantly different in MCI eyes compared with CN individuals. Similar to the central cornea, DC field area (Figure 3B: MCI: 118.4 ± 11.2 versus CN: 75.7 ± 28.4 μm^2^, *p* = 0.04) and DC perimeter (Figure 3C: MCI: 49.0 ± 2.4 versus CN: 38.1 ± 7.8 μm, *p* = 0.02) were greater in MCI participants relative to CN controls. The “per cell” analysis of DC field area in the inferior whorl (Figure 3D) showed that, as a population, corneal DCs from CNs had smaller field areas than cells from individuals with both MCI and AD (*p* = 0.0002). In addition, corneal DCs in the whorl region of MCI participants had lower circularity (Figure 3E: MCI: 0.56 ± 0.03 versus CN: 0.65 ± 0.05, *p* = 0.02), less roundness (Figure 3F: MCI: 0.49 ± 0.03 versus CN: 0.62 ± 0.08, *p* = 0.04), and a higher aspect ratio (Figure 3G: MCI: 2.52 ± 0.19 versus CN: 1.92 ± 0.29, *p* = 0.01), relative to CNs; these findings indicate that the corneal DCs of MCI participants had a larger and more elongated shape.

## Discussion

The present study used IVCM to assess the phenotypic characteristics of corneal epithelial DCs, and corneal nerve architecture, in individuals with MCI and AD. We show, for the first time, that corneal DCs have an altered morphology (i.e., larger average field area and perimeter) in individuals with MCI; this morphological difference was evident in both the central and inferior whorl (mid-peripheral) regions and occurred in the absence of altered DC density. A “per cell” analysis of DC field area, which considered the morphological characteristics of DCs as a population, also showed that the DCs from individuals with MCI and AD had larger field areas than cells from CN individuals. We found nerve morphometric parameters (i.e., density and thickness) in the central cornea, and nerve density in the inferior whorl region, to be similar in the three clinical groups (CN, MCI, AD). The finding of altered corneal DC morphology in MCI, in the absence of overt sensory nerve degeneration, supports the concept that an altered corneal immune cell phenotype may precede peripheral nerve degeneration in CNS disease. These data also provide rationale to investigate whether corneal immune cell characterisation may provide a novel, non-invasive strategy for identifying MCI, prior to the development of clinically significant CNS neuropathology.

*In vivo* confocal microscopy is an ophthalmic imaging technique that provides clinical capacity to directly and non-invasively examine the cornea at a cellular level (∼ 500× magnification), with resolution almost comparable to *ex vivo* histochemistry. Laser-scanning IVCM of the cornea has been used to study the effects of a range of systemic conditions on peripheral nerve integrity, most notably peripheral neuropathy in diabetes mellitus. In diabetic peripheral neuropathy, image analysis of corneal small nerve fiber architecture has utility for detecting (Messmer et al., 2010; Petropoulos et al., 2014; Pritchard et al., 2014; Misra et al., 2015), stratifying the severity (Tavakoli et al., 2010; Yan et al., 2020) and assessing the efficacy of therapeutic interventions (Brines et al., 2014). Notably, the comparisons of nerve parameters in the inferior whorl region has been identified to have greater sensitivity for detecting progressive corneal nerve loss, compared with the central cornea (Ferdousi et al., 2020).

There has also been interest in understanding how corneal nerve analysis can provide insight into the peripheral manifestations of CNS disease. IVCM has been used to characterize corneal nerve abnormalities in individuals with multiple sclerosis (Bitirgen et al., 2017; Mikolajczak et al., 2017; Petropoulos et al., 2017), Parkinson’s disease (Podgorny et al., 2016; Misra et al., 2017), amyotrophic lateral sclerosis (Ferrari et al., 2014) and, most recently, in patients with MCI and dementia (Ponirakis et al., 2019). Using IVCM, Ponirakis et al. (2019) evaluated the central corneal nerve characteristics of a heterogeneous cohort of 26 participants with dementia, including individuals with AD and vascular dementia. These authors reported fewer nerve fibers in the central cornea of people with both MCI and dementia, relative to healthy controls (Ponirakis et al., 2019). The researchers also observed that when adjusted for confounders, central corneal nerve fiber measures were significantly associated with cognitive function and functional independence. Reduced corneal sensitivity and altered tear production have also been described in patients with AD (Ornek et al., 2015). In our study, we found several key corneal nerve parameters, including nerve density and thickness, to be similar in the three clinical populations (CN, MCI, AD). We did, however, observe a trend toward lower nerve morphometric parameters (i.e., nerve fiber length, fiber density, and branch density) in MCI and AD patients. These data are broadly consistent with the findings reported in the Ponirakis et al. (2019), although the absence of a significant result in our study most likely relates to the conservative sample size, with insufficient statistical power to detect inter-group differences for these outcome measures. The finding may also be due to differences in the severity of CNS neurodegeneration in the study populations. In particular, we acknowledge that three out of the five CN participants in the current study were positive for Aβ on PET. A larger study, involving a greater spectrum of clinical phenotypes, would assist with separating PET findings and clinical (cognitive) status, and how these features relate to corneal nerve parameters.

The most striking, and novel, finding in the present study was that corneal epithelial DCs have an altered phenotype in MCI, in the absence of abnormal DC density. In addition, there were no significant inter-group differences for most DC parameters between control and AD participants. These findings are important as DC morphology is regarded as an indicator of the immunological status of the cornea (Jiao et al., 2019). In healthy human eyes, corneal DCs imaged with IVCM typically do not have obvious dendrites and are considered to exist in a relatively immature state (Mastropasqua et al., 2006). In the present study, the two-dimensional analysis of corneal DCs in MCI eyes revealed an enlarged field area and perimeter, indicative of a greater overall size and more complex dendritic arborisation. DC enlargement has been suggested to be a sign of cellular maturation, activation and antigen-presentation capacity (Mastropasqua et al., 2006). Enlarged corneal DCs have been described in a range of conditions characterized by chronic systemic inflammation, including autoimmune diseases, such as graft versus host disease and Sjögren’s syndrome (Kheirkhah et al., 2015). Of direct relevance to MCI and AD is a potential link between systemic inflammatory processes and cognition (Tangestani Fard and Stough, 2019). For example, in the Sydney memory and aging study cohort, individuals with MCI had relatively higher serum levels of the pro-inflammatory cytokine tumor necrosis factor-alpha than CN participants (Trollor et al., 2010). Another cross-sectional study in older adults reported an association between higher blood C-reactive protein (CRP) and interleukin-6 levels and poorer cognitive performance on the Modified Mini-Mental State Examination (Yaffe et al., 2003). Furthermore, it has been reported that in individuals with ankylosing spondylitis, high levels of serum CRP were associated with a greater number of enlarged corneal DCs, when compared to healthy controls (Marsovszky et al., 2014). These findings are consistent with the hypothesis that inflammation is a precursor to the development of neurodegeneration in AD (Metti and Cauley, 2012). It is also of note that the acute-phase protein CRP has been found to be lower in individuals with established AD (O’Bryant et al., 2010), suggesting that the pro-inflammatory overlay may be related to disease progression rather than the disease endpoint. Our finding of altered DC morphology in MCI, but not AD, patients is consistent with this concept.

We note that the quantified corneal DC field area in control participants was relatively smaller than that described in another study that used a similar IVCM methodology (Kheirkhah et al., 2015). This difference may relate to two key factors. First, with respect to methodology, Kheirkhah et al. (2015) quantified DC morphological parameters for “the 10 most representative cells in three images for each eye.” The authors did not describe their criteria for selecting the “representative cells,” which creates the potential for a biased sample to have been analyzed. In contrast, in the present study we quantified 20 unique corneal DCs per participant. To minimize any potential bias in cell selection, cells were consecutively quantified from the top left hand corner of randomly selected images, until the required number of samples was achieved. Between four and eight separate non-overlapping central corneal image regions were required to achieve the nominated number of DCs across the study population. Second, the “control” populations in the Kheirkhah et al. (2015) study and the present study are not equivalent. Kheirkhah et al. (2015) recruited 20 control participants (mean ± SD, age: 57.9 ± 7.6 years) “with a clear healthy cornea without vital staining and normal tear function tests.” In the current study, we recruited five control participants, also “without clinically significant ocular surface pathology,” but who were significantly older (mean ± SD, age: 74.0 ± 3.5 years). At present, few studies have analyzed DC morphological parameters in human populations using IVCM. As such, the effect of aging on corneal DC morphology is unclear, but the differing ages of the study populations may be a contributory factor to the inter-study differences.

Of direct relevance to the present findings, our research group have also recently discovered that corneal sensory nerves and epithelial DCs are altered in the rTg4510 mouse model of tauopathy, with temporal changes observed in association with aging (Jiao et al., 2020). Specifically, in this experimental model of CNS tauopathy, changes to corneal DC morphology were evident prior to the onset of corneal nerve axon pathology, which occurred in later stages of the disease. Based upon our current study’s findings, we propose that a similar pattern of change may occur in human CNS disease, whereby corneal immune cell activation (indicated by DC enlargement) may be a relatively early indicator of CNS pathology in individuals with MCI that occurs prior to peripheral sensory nerve degeneration. Longitudinal studies, involving a sufficient sample size, are warranted to investigate this hypothesis.

In conclusion, this is the first clinical study to report morphological differences in corneal DCs in people with MCI. These differences were evident in the central and mid-peripheral cornea, and occurred in the absence of a difference in DC density or marked sensory nerve degeneration. The larger corneal DC field area and perimeter in MCI eyes are consistent with an immunologically activated cell state. These findings provide rationale for evaluating the diagnostic accuracy, including sensitivity and specificity, of corneal imaging of epithelial DC parameters as a disease marker in larger clinical populations MCI and AD.

## Data Availability Statement

The raw data supporting the conclusions of this article will be made available by the authors, without undue reservation, subject to receiving an ethics amendment approval for data sharing.

## Ethics Statement

This study received ethics approval from St Vincent’s Hospital (Melbourne) Human Research Ethics Committee (HREC) (Ref: HREC-A 028/06), was endorsed by the CSIRO Health and Medical Research Ethics Committee (RR 5/2018) and was prospectively registered with the University of Melbourne HREC (ID: 1852006). All participants provided written informed consent to participate.

## Author Contributions

CD, SF, RJ, CF, CM, YK, and LD conceived and designed the study. CD and LD performed the ocular examinations. CD, HJ, JL, and LD analyzed the data, with intellectual input to the analytical approaches from HC. All authors contributed to the interpretation of data for the study, drafting of the manuscript, agreement to be accountable for all aspects of the work, and approved the final submitted version of the manuscript.

## Conflict of Interest

The authors declare that the research was conducted in the absence of any commercial or financial relationships that could be construed as a potential conflict of interest.
