# Supplementary material for: Morphometric Changes to Corneal Dendritic Cells in Individuals With Mild Cognitive Impairment
Source: Front Neurosci. 2020 Dec 9;14:556137. doi: 10.3389/fnins.2020.556137 (PMC7755610; doi:10.3389/fnins.2020.556137)
Supplement: Supplementary file 1 [file Data_Sheet_1.pdf]

# *Supplementary Material*

**Figure S1. Representative IVCM images of the central corneal region, for each study participant.**

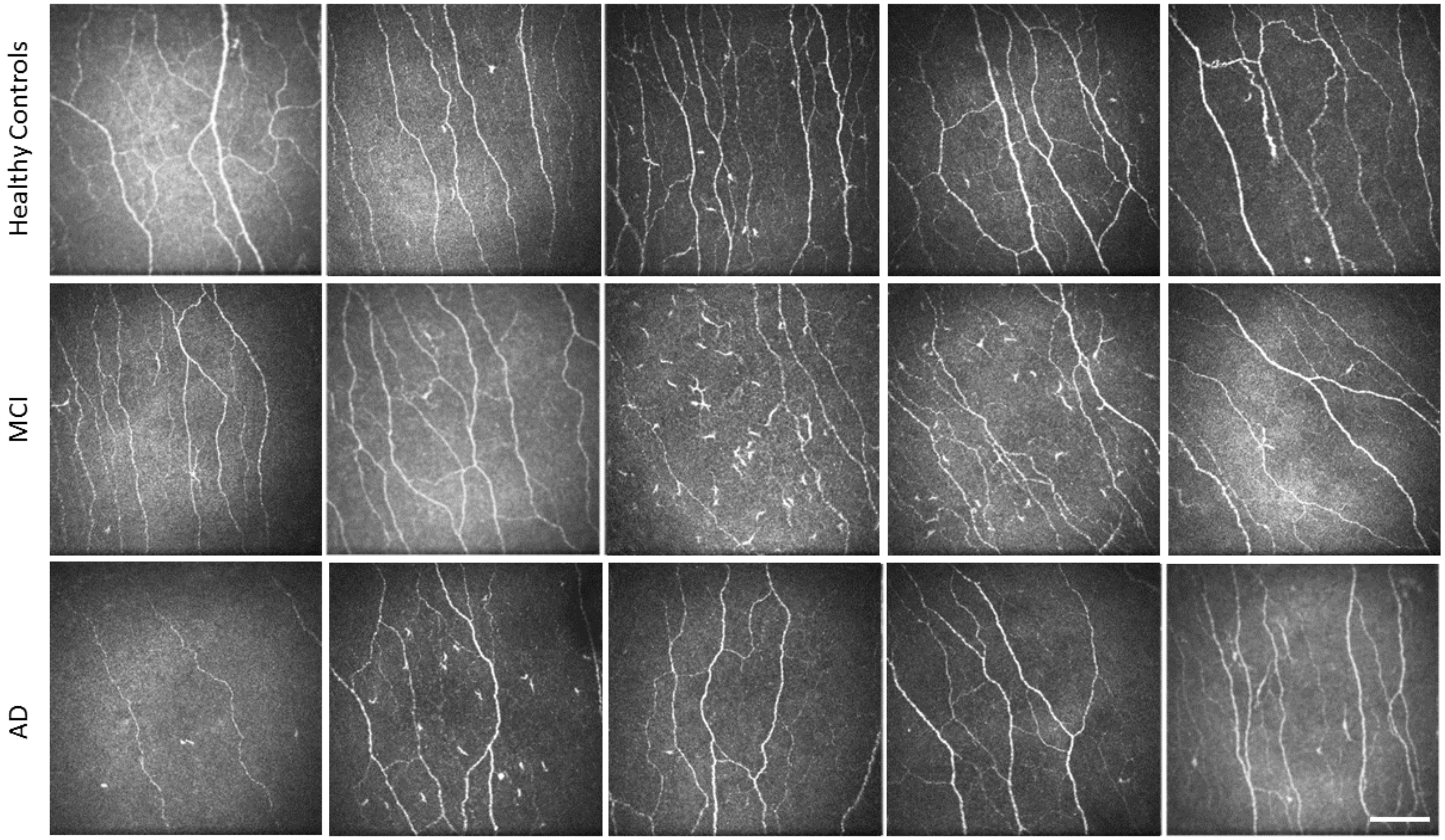


Representative central corneal IVCM images for each study participant, for healthy control (upper row, n=5), MCI (middle row, n=5) and AD (lower row, n=5) participants. Scale bar = 100µm and applies to all images.

**Figure S2. Additional central corneal dendritic cell parameters**

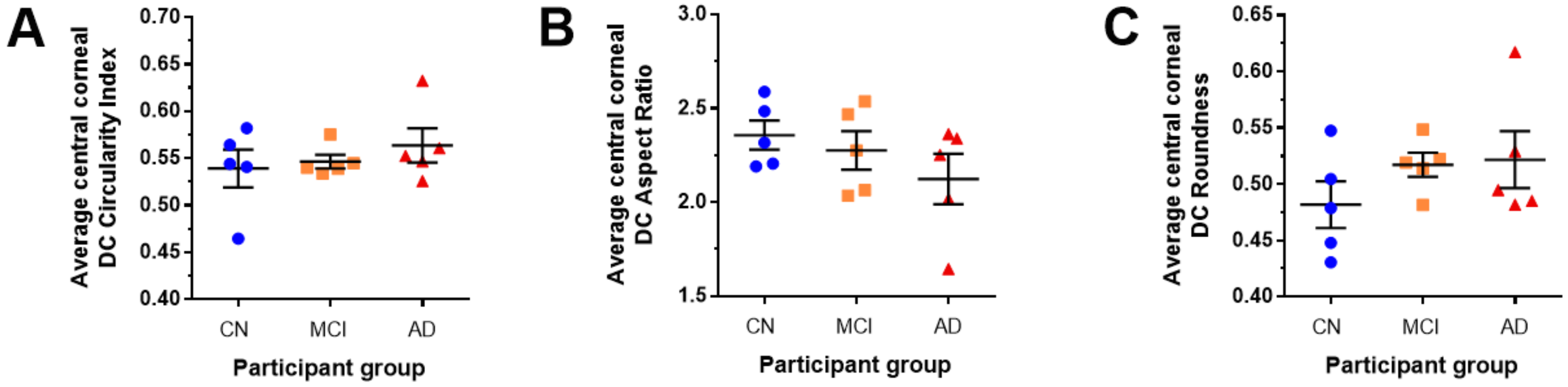


Central corneal DCs had a similar shape with respect to circularity **(A)**, aspect ratio **(B)** and roundness **(C)** in cognitive normal (CN), mild cognitive impairment (MCI) and Alzheimer's disease (AD) participants; none of the comparisons were statistically significant ($p>0.05$ for all comparisons).

**Figure S3. Representative IVCM images of the corneal inferior whorl region, for each study participant.**

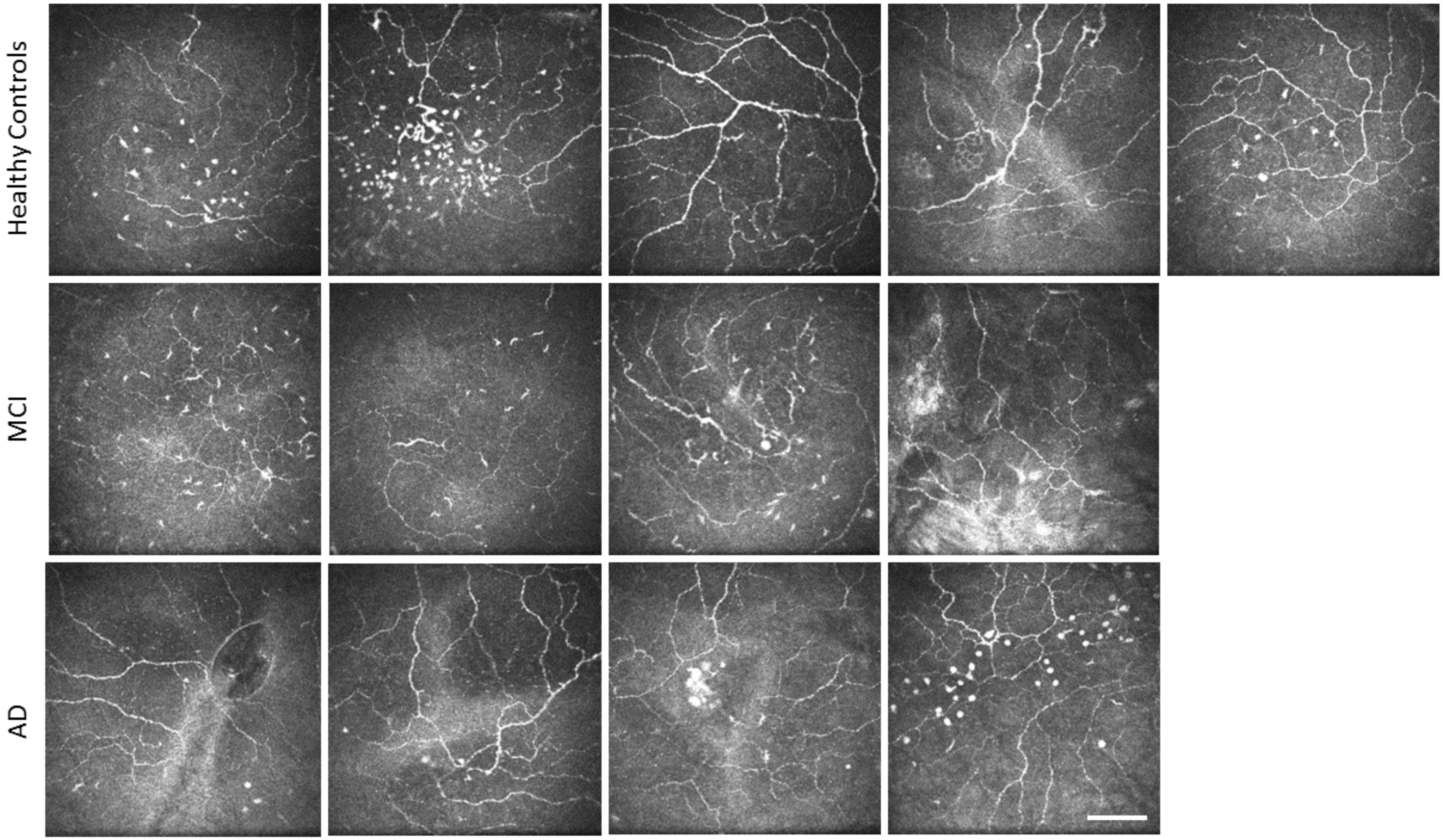


Representative corneal IVCM images of the inferior whorl region for each study participant, for healthy control (upper row, n=5), MCI (middle row, n=4) and AD (lower row, n=4) participants. Scale bar = 100µm and applies to all images.

**Table S1. Summary of participants' concomitant medications**

| MEDICATION CATEGORY | CN (n=5) | MCI (n=5) | AD (n=5) |
|---|---|---|---|
| **Alimentary** | | | |
| - Anti-diarrhoeal | - | 1 | - |
| - Hyperacidity, reflex and ulcers | 3 | 2 | 1 |
| **Anti-infective** | | | |
| - Combination anti-viral (hepatitis C) | - | 1 | - |
| **Cardiovascular** | | | |
| - Anti-coagulant | 2 | 2 | 3 |
| - Anti-hypertensive | 2 | 4 | 3 |
| - Hypolipidaemic | - | 2 | 2 |
| **CNS agents** | | | |
| - Antidepressant | - | - | 1 |
| - Anticovulsant | - | 1 | 1 |
| - Cholinesterase inhibitor | - | - | 4 |
| - Sedatives/hypnotics | - | - | - |
| **Endocrine and metabolic** | | | |
| - Agents affecting calcium and bone metabolism | - | - | - |
| - Gonadal hormones | 1 | - | 1 |
| **Genitourinary** | 1 | 1 | |
| Musculoskeletal | | | |
| - Agent for gout or hyperuricaemia | - | 1 | 1 |
| - Muscle relaxant | - | 2 | - |
| - NSAID | - | 2 | 3 |
| - Simple analgesic | - | 1 | 2 |
| **Respiratory** | | | |
| - Preventative aerosols and inhalations | - | 1 | 1 |
| **Vitamin supplements / complementary medicines** | - | 1 | - |
| - Curcumin | - | 1 | - |
| - Ginkgo biloba | 1 | 1 | 1 |
| - Glucosamine | 2 | - | 2 |

| | | | |
|---|---|---|---|
| - Magnesium | 1 | - | 2 |
| - Multivitamin | 2 | - | - |
| - Omega-3 fatty acids | 1 | - | - |
| - Souvanaid | - | 1 | 4 |
| - Vitamin B12 | - | - | 1 |
| - Vitamin C | 1 | - | - |
| - Vitamin D | 3 | - | 1 |

Data are presented counts for each categorical variable.

Legend: AD, Alzheimer's disease; CN, cognitive normal; CNS, central nervous system; MCI, mild cognitive impairment; NSAID, non-steroidal anti-inflammatory drug
